# When and Why Cats Are Returned to Shelters

**DOI:** 10.3390/ani13020243

**Published:** 2023-01-09

**Authors:** Vivian Mundschau, Malini Suchak

**Affiliations:** Department of Animal Behavior, Ecology, and Conservation, Canisius College, 2001 Main St., Buffalo, NY 14208, USA

**Keywords:** animal shelter, cat, felis catus, returns, foster

## Abstract

**Simple Summary:**

Shelters hope that when cats are adopted, they are moving into their “forever homes”; however, a non-trivial number of cats are returned to the shelter after adoption. It is helpful for shelters to understand why, as cats who are returned impact the ability of the shelter to take in new cats and can negatively impact the returned cat’s welfare. We looked at which cats might be at a higher risk of being returned and what reasons the owners gave for returning their cats. We also compared whether cats were returned soon after adoption (within 30 days) versus over a longer period of time (more than 30 days to 4 years after adoption). We found that the reasons cats were returned shortly after adoption were focused on specific issues with the cat, such as behavioral problems or conflicts with other pets. In contrast, cats who were returned in the long term often had to do with the owner, such as major life changes (births, deaths, illnesses, children) or the cost of owning the pet. Our findings suggest that shelters might need to consider different types of strategies for long-term and short-term returns to reduce the return rates overall.

**Abstract:**

There is considerable research on why cats are initially relinquished to shelters, but much less attention has been given to returns, despite the significant implications for shelter capacity and cat welfare. Furthermore, the structure of many databases fails to account for cats who are returned beyond 30 days, despite this making up a substantial portion of returns. In the current study, we examined common risk factors and reasons for return in a population of 2642 shelter cats. We found that cats who were older at the time of adoption or had a bite history had an increased risk of return, whereas cats that were in foster care prior to adoption had a decreased risk of return. We divided the returns by the time to return (<30 days: short term, >30 days: long term) to examine whether time to return had an impact. Approximately half the cats were returned in the short term. Cats were more likely to be returned for reasons, such as behavior, unwanted, and other pet in the short term and personal reasons, cost, euthanasia, and stray in the long-term return. Strategies to reduce returns should consider different solutions for short and long returns to maximize effectiveness.

## 1. Introduction

Each year in the United States, an estimated 3.2 million cats enter shelters and about 66% are adopted [1]. Of the cats that are adopted, some are returned. Returns are a concern from financial, animal welfare, and community perspectives. When cats are returned, the holding capacity of the shelter is affected. Even small changes in the length of stay of cats in a shelter can have a significant impact on available space in a shelter [2]. Therefore, decreasing the number of cats being returned to a shelter could improve shelter efficiency by freeing up shelter space and resources.

Returning to the shelter is potentially stressful for cats, leading to a compromise in their welfare. While it is impossible to definitively say for all cats if being in a shelter is more stressful than living in a home [3], the type of shelter housing [4,5], density of cats [6,7], cat intake origin [8], and provided enrichment in a shelter [4,9] can impact the level of stress cats experience. Cats who are more stressed in the shelter eat less, lose more weight, and are more likely to develop upper-respiratory infections. A sign of stress can be a change in physical health; 82% of shelter cats lost weight in their first week in the shelter [10]. For owned cats, the transition from home to shelter poses the risk of increased stress compared to stray cats entering the shelter, but it is unknown if this holds true for cats who were adopted and then returned to the shelter [8]. Therefore, returns represent a risk to cat welfare by potentially increasing stress and exposure to disease. 

The impact of returns is not limited to cats, as it also affects the owner’s wellbeing. The act of returning a pet impacts owners negatively, both mentally and emotionally [11]. When adoptions are unsuccessful, resulting in a return, it can impact potential owners’ willingness to adopt from that shelter again depending on the reason for return [12]. Trust in shelters may also be affected, with only 10% of returners adopting again from the shelter they returned a pet to [12]. While there have been concerns that owners may view shelter adoptions as a lesser investment because of the possibility for returns [13], the decision to return a pet is likely not easy for owners. Over 56.6% of pet owners describe returning their pet as “very difficult”. Despite 50.6% reporting the problem that led to return emerging within 24 h after adoption [11], owners still procrastinate surrender, only using the shelter as a last resort [14].

Identifying reasons for returns can be difficult, as shelters may have different wording or categorization of information collected during the return process. In addition, shelters often only record a single reason for a pet’s return. This may not represent the full reason for the return, because upon interview, returners may list more than one reason for the return and may not even mention the reason they listed upon relinquishment [14]. Studies may also categorize types of returns into categories, such as “pet”- or “owner”-related reasons. Grouping reasons is common because it is not unusual for the number of each reason for return to be small, making statistical comparisons difficult. Unfortunately, while this grouping may strengthen statistical analysis, category definitions are not standardized across studies. As a result, trying to find the frequency of a specific type of return within the current literature is not always possible. Researching reasons for returns is also complicated by the fact that it is common for shelter databases to only label an intake as a return if the return is 30 days or less since the adoption [15,16,17]. This format is likely used by shelters because of policies that refund owners if they return their pet within 30 days of adoption; however, this makes it difficult to accurately estimate how many cats return to the shelter after adoption.

Currently, there are three main difficulties in comparing reasons for shelter returns across studies. First, there are few studies that have a description of how long each cat was monitored for return. For example, if a study looked at all the cats adopted over three years, it is important to know if all subjects would have been observed for three years (which would require tracking beyond the 3-year period specified in the study). While some studies have a defined length of observation or return [15,16,17], the description of the return window is absent in many studies. Second, due to the lower number of cat returns compared to dogs in study samples, reasons for cat intake have been combined with reasons for dog intake in several studies [18,19,20,21,22]. This provides limited information about species-specific differences. Third, there has not been a strict separation of reasons for returns after adoption and all types of relinquishment. While some studies have differentiated initial intake from returns [11,16,23], most available research collapses the separation between initial intake and returns to simply reasons for relinquishment [19,21,24,25], likely due, in part, to the intensive process currently required to separate returns from initial intake. Because some shelters only label returns that happen 30 days or less after adoption, finding the total number of returns requires the labor-intensive process of tracking individual cat shelter identification numbers. Therefore, while it is not surprising this task has not been performed frequently, the understanding of shelter cat returns is still limited and more generalized reasons for intake are currently the best sources for common reasons of cat relinquishment. 

Because of the lack of research on returns specifically and the fact that many relinquishment studies combine initial intake with returns, we identified the following as the most common reasons for relinquishment: behavior [11,14,19,20,23,25], owner/personal issues [19,20,23,26], allergies [11,14,20,23,27], owner housing issues [14,19,20,21,28], not enough time to care for pets [24], children problems [11], and other pets [11,16,20,25].

In addition to the time window of return and relinquishment reason, there are factors that indicate an increased likelihood of return, such as younger owners [29], older cats [16,23], factors specific to the owner’s community [30], the presence of another pet in the home [25], children in the home [24], and home renters [21,24,28]. There is also a trend with changes in relinquishment, adoption, and reason for relinquishment in times of social/economic shifts, such as the 2001 terrorist attack [21], the 2008 economic recession [22], and the 2020 global COVID-19 pandemic [26].

It is possible that the timing of return could impact both the reasons as well as any interventions shelters may want to implement. Many shelters categorized a return as a pet being brought back within 30 days of adoption, with any other returns >30 days categorized as a regular intake/surrender [15,16,17]. The current literature on returns and relinquishment varies in the length of time examined, from 2 weeks after adoption [11] to 21 years [19]. A longer time frame may be required to capture the full breadth of returns, as research looking beyond the 30-day window for returns found 57% of adopted cats are returned more than 30 days after adoption [15]. It is possible that the number of returns is underestimated based on a conventional 30-day timescale. This window for returns is also likely too short for most cats to adapt to their new surroundings, as one study found that cats in the home 1–3 months after adoption had similar stress levels to when they were still in the shelter [3]. Since the average length of pet ownership before relinquishment is 18 months [21], a longer window of examination may be warranted in understanding reasons for return, as cats in a short or long time period after adoption may be different and may require different forms of interventions. We also examined the outcome of cats who were returned, for any difference between cats who were returned in the short versus long term.

While there has been research on how factors after adoption impact the return of cats [21,22,24,25,26,28], less emphasis has been given to features in the shelter prior to the cat’s initial adoption. This may be significant, as shelter housing type may affect stress levels and behavior [5,6,7,31]. This could lead to differences in adoption choices and satisfaction with adopted pets, as adopters look for friendliness toward the adopter, “happy” cats, and playfulness [32]. Suchak and Lamica [17] found that in a cohort of cats matched for features, such as age and breed, cats initially placed in group housing were more likely to be returned than cats placed in single-cat housing. It is possible that potential owners may view cats in group housing as more sociable with other cats, resulting in more returns due to problems with other pets in the home [17], but the sample of returned cats was very low, which made it difficult to identify any patterns based on reason for return. Since Suchak and Lamica [17] categorized cats based on their intake location, some cats may have been moved to other locations, such as offsite or foster care, prior to being adopted. Therefore, in addition to looking at demographic factors affecting likelihood of return, we will also be investigating to see if adoption location affects likelihood of returns, as well as the reason cats are returned relative to their adoption location.

There are four goals in the current study: (1) identify the most common reasons for cat returns after adoption, (2) collect data on the various characteristics of the cats (intake age, adoption location, bite history) to see if any commonly noted characteristics are predictive of returns, (3) compare the time of returns (short vs. long) with the reason for return and characteristics of the cat, and (4) compare the outcome of cats who are returned in the short and long term. 

## 2. Materials and Methods

### 2.1. Setting

Data were collected at a large, managed-intake shelter in the Northeastern United States from January 2014 to December 2016. The shelter uses a waiting list when at capacity and is open admission at other times of the year. While at the shelter, cats are housed in a number of different settings or locations, including single metal cages (0.38–0.85 m^2^ of floor space) or group rooms (3.06–5.41 m^2^ of floor space, median density of 1.02–1.80 m^2^ per cat). Single cages typically have a bed, food dish, toys, and litter box, whereas group housing was located in a large room with benches, climbing structures, crates, beds, and toys. In previous work [17], we found that cats often moved through more than one housing setting and that in a matched cohort of cats adopted from group housing and caging, cats from group housing were more likely to be returned. We did not include any offsite housing in our previous analysis; however, cats were frequently sent into foster care or sent to one of many offsite locations located in pet supply stores or malls. Offsite housing was a combination of group and single caging. In all settings, dry food and water were given ad libitum and wet foot was provided twice per day. As this study did not use any live animals, only data, it was exempt from Institutional Animal Care and Use Committee approval.

### 2.2. Subjects

Over the course of the 3-year period, 2642 cats were brought into the shelter for the first time and adopted. We only included cats for whom this was their initial intake into the shelter, as determined using the PetPoint database of electronic records to individually look up the unique identification numbers of cats. Initial intake was defined as when a cat is brought to a shelter for the first time. We defined a return as when a cat was brought back to a shelter after adoption. As we used the ID numbers to determine returns, it should be noted that some cats were returned by individuals other than the adopters on record. If a cat was returned more than once, we only collected data on the first reason for and outcome of the first return and only noted the total number of returns.

We also recorded each cat’s age at adoption by subtracting their date of birth (a date estimated upon intake) from their adoption date. Only cats greater than 1 year of age upon intake were included in the study, as this corresponded to the sample in our previous study [17]. We also recorded whether or not a cat had been noted as having bitten someone while at the shelter or right before their return to the shelter. This information was found either at the top of the cat’s profile, in the medical report, or in the notes. Although there are any number of behavioral problems that could lead to return, the clear shelter procedures and logging methods for bite cases, required because of the risk of rabies, made this a prime candidate for identifying behavioral risk factors. 

Reasons for return were logged by shelter staff as a single entry in an open text box; thus, the admissions staff could freely write down any single reason for return. We collapsed these into 11 different categories based on previous research, also trying to avoid any categories with only a few cats:

- Euthanasia: The cat was returned by the owner to be euthanized. Note that this is independent from the outcome of the return as the shelter evaluated these cases independently; some cats who were returned to be euthanized were treated and later adopted out.
- Stray: The cat was adopted from the shelter but later brought in as a stray by a community member. The shelter was able to attach the cat to a previous record using their microchip.
- Medical: The cat was returned for medical treatment or because they were ill. Note that if the person cited the cost of medical care specifically, rather than a medical issue itself, that was included in “cost” below.
- Behavior: Behavior was commonly cited verbatim as the reason, but we also included house soiling and biting in this category.
- “Unwanted”: These cats were listed verbatim as unwanted by the adopter, with no further explanation.
- “Other pet”: This was listed verbatim, typically with no further explanation. This reason is commonly cited in other studies of this type [15,16,20,25].
- Allergic: The owner indicated someone in the household was allergic to the cat.
- Housing: Owners who cited housing issues included references to eviction, moving, and problems with landlords.
- Cost: Some owners specifically cited cost, while others noted medical costs.
- Personal issues: There was a wide scope of personal issues, ranging from family changes, such as pregnancy, new baby, and divorce, to the owner being ill or deceased, to issues, such as travel or no time.
- Other: This included any category with fewer than 3 cats, including transfer, cats seized in cruelty cases, and cats returned because the owner had “too many”.

We grouped type of outcome into three categories: adoption (cat goes to a new family), euthanasia (cat is put to sleep for humane, medical, or behavioral reasons), and transfer (cat is moved to a different shelter). Data are available as a supplemental file S1.

### 2.3. Analysis

All data were analyzed using R version 3.6.0 (R Core Team, 2019). We first analyzed the dataset as a whole to examine risk factors for return, including age at intake, bite history at the shelter, and adoption location. Categorical variables (bite history and adoption location) were analyzed using a chi-square test. Age, as a continuous variable, was analyzed using a Wilcoxon rank-sum test or a Kruskal–Wallis test depending on the number of levels of the independent variable. To determine whether there were significant differences between short-term (<30 days) and long-term returns, we used Fisher’s exact tests because several cells had fewer than 5 individuals. Risk ratios were calculated to determine the magnitude of the difference using gigacalculator (https://www.gigacalculator.com/calculators/relative-risk-calculator.php, accessed on 17 November 2022). All alpha levels were set at *p* < 0.05.

## 3. Results

**Figure 1:**
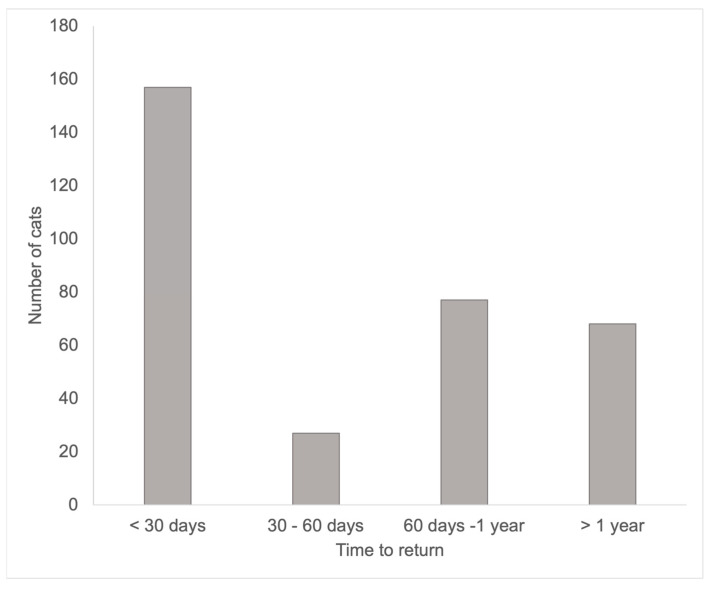
Number of cats returned to the shelter over time.

Overall, out of 2642 cats who were adopted, 309 (11.70%, 140 males, 168 females, 1 unknown sex) were returned within 4 years of their adoption (Figure 1), with the majority of cats (283, 91.59% being returned once) and a small minority being returned twice (21, 6.80%) or three times (5, 1.61%). Out of the cats who were returned, 263 (85.11%) were adopted, 11.97% were euthanized, and 2.91% were transferred to another shelter or rescue organization. Approximately half of the returned cats were brought back to the shelter within 30 days (short-term returns: 155 cats, 50.16%). This means that the remaining half were returned beyond the noted 30-day return window (long-term returns: 154 cats, 49.84%). The vast majority of cats was adopted following a short-term (*n* = 141, 88.68%) and long-term (*n* = 122, 77.70%) return. However, there was a significant difference in outcomes for short- versus long-term returns (Fisher’s exact test, *p* = 0.0003). Cats that were returned in the short term had a significantly higher probability of being adopted than cats in the long term (Table 1). Conversely, cats returned long term had a significantly higher probability of being euthanized than cats returned in the short term.

Of the 11 reasons for return, the most common reason was behavior (25.56% of returns), followed by personal (13.59%), other pet (10.36%), medical (8.73%), allergies (8.41%), cost (8.10%), housing (7.44%), and stray (6.47%). The remaining categories accounted for less than 5% of the total returns each: euthanasia, unwanted, and other. There was also a significant difference in reasons given for return between short-term and long-term returns (Fisher’s exact test, *p* = 0.0005). Cats had a significantly higher probability of being returned in the short term for behavioral problems, issues with other pets in the household, and being unwanted (Table 2). Cats had a significantly higher probability of being returned in the long term for other issues, including the owner’s personal issues, cost, stray, and euthanasia.

Although the median age at adoption was only 3.42 years for cats who were returned (as compared to 3.04 years for cats who were not returned), age at adoption significantly impacted the likelihood of return (W = 3,156,774, *p* = 0.0008). There was no significant difference in age at adoption on whether the cat was a short- or long-term return (W = 13,069, *p* = 0.15). There was a significant difference for reason for return based on age at adoption (Kruskal–Wallis *χ*^2^ = 18.753, df = 10, *p* = 0.04). Cats who were returned for euthanasia had the highest median age at adoption (7.59 years), followed by cats returned for “other” reasons (6.45 years). The remaining reasons all had a median age of between 3 and 4.1 years. There was a trend, but no significant relationship between age at adoption and adoption location among returned cats (Kruskal–Wallis *χ*^2^ = 7.62, df = 3, *p* = 0.05), with foster cats having the highest median age at adoption (5.01 years), followed by group-housed cats (4.03 years), caging (3.10 years), and offsite (3.08 years).

**Figure 2:**
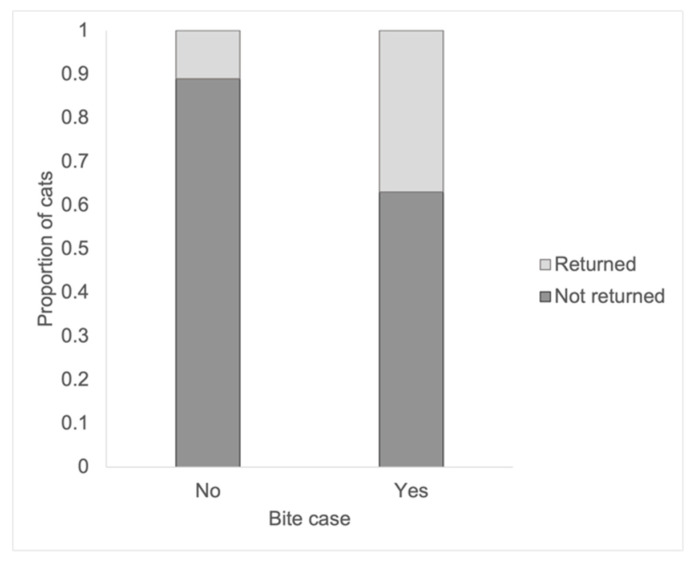
Proportion of cats returned and not returned by whether they were classified as a bite case while at the shelter. Significantly more cats with a history of biting were returned as compared to cats without a bite history.

A bite history while in the shelter also significantly impacted the likelihood of return (*χ*^2^^2^ = 51.98, df = 1, *p* < 0.0001, Figure 2), with cats who were a bite case while in the shelter having a 4.37-times higher probability of being returned (Risk Ratio (RR): 4.37, 95% CI: 1.88–6.65, Z = 6.90, *p* < 0.0001). An equal number of cats with a bite history were returned in the short term (*n* = 16) and long term (*n* = 16; Fisher’s exact test, *p* = 1.00). While the majority of cats with a bite history were returned for behavior (*n* = 18, 56.25%), cats with a bite history were returned for every reason except unwanted and other.

Adoption location also significantly impacted returns (*χ*^2^^2^ = 18.63, df = 3, *p* = 0.0003). Cats adopted from group housing had the highest rate of return (16.61% of group adoptions), followed by cats in single caging (13.42%) and cats adopted at offsite locations (10.99%). Cats adopted from foster care had the lowest rate of return at only 7.02%. There was a significantly different pattern of short-term versus long-term returns based on adoption location (Fisher’s exact test: *p* = 0.002). Just over half the cats adopted from single caging (50.96%) and a majority of cats adopted from group housing (60.78%) and offsite (53.6%) were returned in the short term. In contrast, only 13.79% of cats from foster care were returned in the short term and a strong majority from foster care (86.20%) was returned in the long term. Finally, when looking at reasons for return by adoption location, for all the locations, behavior and personal reasons were among the top-four reasons for return. For caging, group housing, and offsite, other pet was another top reason, but zero cats were returned due to other pets when adopted from foster care. Euthanasia and cost were in the top-four reasons for foster care, but none of the other locations, which is likely due to the fact that a disproportionate number of foster returns were long term. Finally, allergic was a top reason in foster cats and offsite adoptions, but not in the other two locations.

## 4. Discussion

Similar to previous studies, we found behavior [14,15,16,19,20,23,24,26], personal [20,23,24,26], other pets [15,16,20,25], medical [15,30,33], allergies [14,15,16,20], cost [26,30], and housing [14,15,19,20,30,34] to be common reasons for return. We also found stray to be a reason for return despite this not being listed by other studies in the past. The reason for this is likely because we classified a return as any pet returning to the shelter they were adopted from, whereas other studies may have only included cats who were returned by the owner who originally adopted them. While the number of cats returned as strays is not very large, it may represent a source of cats returning to the shelter that needs further consideration. 

We anticipated that cats housed in group housing might be returned more based on earlier work by Suchak and Lamica [17], and while the difference is not statistically significant, group-housed cats are returned at a higher rate than caging, offsite, or foster cats. Unexpectedly, cats adopted from foster care were less likely to be returned than cats from any other adoption site. There are two possible reasons for this difference. First, in the work from Suchak and Lamica [17], location was logged based on the cat’s first location in the shelter compared to this study where location was recorded as the last location of a cat in the shelter. Second, Suchak and Lamica [17] only compared two housing locations, as opposed to the current study, which had four categories of locations. The main purpose of foster care is for cats who have medical or behavioral issues; therefore, it is unexpected that cats from foster care would be returned less overall, as behavior and medical reasons were some of the most common reasons for return in our study. 

We found medical, as a reason for return, was more common in short-term returns than long-term returns, but the difference was not statistically significant. Previous literature supports the trend that medical issues are common in the first few weeks of pet ownership. When owners were interviewed one week and one month after adoption, 51.9% of owners reported their dogs or cats had at least one unresolved medical problem after one week and 10.3% of dogs and cats still have at least one unresolved medical problem one month after adoption [35]. There were fewer foster cats returned in the short term than any other location. Since one of the main reasons cats go into foster care is for medical reasons, it may be that potential adopters are more informed about the cat’s current medical issues, resulting in fewer returns. This would explain why no cats adopted from foster care and returned in the short term cited medical issues as the reason.

Perhaps unsurprisingly, we found cats with a bite case history have a 4.37-times higher probability of being returned. While specific data on bites as a reason for return are low, cats being returned for behavioral reasons is commonly cited [11,14,19,20,23,25,26]. Therefore, it is not surprising that cats who have a history of biting are returned more frequently. Because regulations often require the recording of bite cases in the United States to monitor for rabies, it is a clear, undesirable behavior tracked by many shelters. For this reason, bite cases could be a way for shelters to pinpoint cats who are at higher risk of return and provide additional education to potential owners on the special needs of the cat. That being said, not all returned cats who had a bite history were returned because of behavior. This could be because returns often cannot be summed up to a single reason [14]. It could also be because owners do not want to harm the odds of their pet being adopted by mentioning behavior problems or behavior simply was not the main reason for return. 

It is currently unclear why adoption location impacts a cat’s likelihood of being returned to the shelter. It may be that foster cats have more time to adjust to the home before owners feel pressured to return the cat. However, this would not completely explain this trend, because while some of the cats in foster care were adopted by their foster parents, this was not always the case. It could be that cats are less stressed in foster homes and more easily transition to their permanent homes but evidence of the home being less stressful than the shelter, especially in the first few months in the home, is lacking [3,36]. Another factor to consider is the foster parents themselves. It is possible that foster parents who become adopters may be different from other adopters in some characteristics, leading them to return fewer cats. It was beyond the scope of this study to track how many foster parents adopted their foster cats and it is unknown if those who start as foster parents and adopt are less likely to return their cats. It should be made clear that while fostering seems to result in fewer returns, increasing the number of cats housed in foster homes may not be practical or even possible. Foster-care programs are intensive for the shelter in terms of finding foster parents, follow up, and legal considerations and some shelters may not have the resources to increase the scale of their foster programs [37,38].

Currently, time to return is not a factor that is purposefully measured in studies about shelter-cat returns. We were only able to find one other study that specifically compared between short and long returns but they were still relatively short term (fewer than 30 days, between 30 and 60 days, or greater than 60 days [15]). We took a broader look at returns, including data four years after each cat had been adopted. Similar to Hawes et al. [15], who found that 47% of cats were returned in 30 days or less, we found roughly half of returns (50.16%) happened within 30 days after adoption. We found the reason for returns varied significantly between short and long returns. Short-term return reasons seem to be issues related to the pet adapting to their new home (behavior, other pets in the household, and being unwanted), whereas long-term returns are more linked with a change in the owner’s situation (owner’s personal issues, cost, stay, and euthanasia). This indicates that to decrease the number of shelter returns, different approaches should be taken to reduce short- versus long-term returns. 

The benefits of understanding the reasons cats are returned, as well as the timescale of those reasons, become clear in the creation of safety-net programs. Safety-net programs are systems that support pet owners to keep ownership of their pets through assistance with basic needs, accessible veterinary care, and accessible behavioral care [34]. The needs of each community will vary [30] and may change over time, especially during social/economic shifts [21,26,28], but by knowing the most common reasons for return, as well as the time frames owners are most likely to return in, shelters can target their support to those individuals. When attempting to reduce short returns, factors related to the pet should be considered, such as behavior counseling, more in-depth information on pet introductions, and clear expectations of pet ownership. For long-term returns a different approach is needed, one that addresses the needs of the owner. Shelters can direct owners to low-cost veterinary care, pet-friendly housing, or offer basics, such as pet food and litter. These types of programs are critical because when services, such as low-cost veterinary care, are hard to access, owners may resort to shelters [39]. This form of safety-net programing may not only reduce returns, it can also save the shelter money if the support provided to owners is less than the cost of processing and caring for a returned cat [40]. The structure of shelter intake may also be important to consider when attempting to decrease returns. Shelters with a controlled intake have the advantage of being able to offer alternatives to owners when they are considering returning their pets [41]. This gives shelters a gap of time to determine if intervention should be attempted, and if so, what kind.

There are cases when owners temporarily cannot take care of their pets (homelessness, short-term prison time, getting out of a domestic abuse situation, hospitalization, etc.). Currently, there are very few options for those who cannot take care of their pet and for many, returning the animals to the shelter might be the only option or the last resort [14,28,34,40]. One possible alternative is temporary foster programs. Such programs could help keep more owners and pets together, decreasing the number of pets being returned to the shelter. This issue is not only an animal-welfare issue, but a human-welfare issue as well. Having to leave behind a pet can delay individuals from exiting a dangerous relationship. One foster-care program specifically tailored to the needs of a women’s shelter has seen great success for both owners and pets [42].

The effort to reduce returns should not be the sole responsibility of shelters. Veterinarians are in a unique position, as they may be the only professionals who owners see regularly. Veterinarians can play a vital role in education on socialization practices, problem behavior solutions, and low-cost spay and neuter programs [43]. Such programs help owners recognize normal cat behavior and learn ways to positively interact with their cats. Strengthening the pet–owner bond in the early days of adoption may decrease the number of returns [44]. There also needs to be a conscious effort to increase the number of rentable properties that allow for pets, as the lack of pet-friendly housing continues to be an issue for many owners [21,28].

Finally, our study provides important reference data on baseline rates of return in a shelter population outside of any major social or economic world events. Our data collection period started 6 years after the 2008 recession and ended 4 years prior to the COVID-19 pandemic. As there is considerable interest in the impact of the pandemic on pet relinquishment [26], and particularly returns of pets adopted during the pandemic, our dataset provides an important baseline against which new data can be compared.

### Limitations

There are a few limitations to the current study. One of the major limitations was that we were only able to use data from one shelter, and our findings could be limited in their application to other shelters. Further, relying on owner reports of a single reason for return is a weakness, which may not capture the full scope of why the cat was returned [14]. Still, shelters can only act upon the data they have available, and this study provides a framework for identifying reasons for cat shelter returns, which can be used to analyze other shelters’ data. Going forward, a study comparing the reasons for return in short- vs. long-term returns across multiple shelters would greatly expand understanding of the reason for return relative to the time of the return.

We note also that due to the longitudinal nature and the necessary lag needed to manually collect these data, the data presented in this study are already several years old. While this was necessary due to the fact that we examined data for up to 4 years after adoption, this does have implications for applying the results to the current state of sheltering. In particular, given the impact of the COVID-19 pandemic [26], it may be necessary to replicate this study in future years.

Another limitation was that we only followed the first return of cats. Further work needs to be conducted to understand if the reasons for returns remain consistent across multiple returns and what characteristics are common amongst chronically returned cats. While our sample of cats who were returned more than once was too small to analyze, it is possible that with a larger sample, more common themes could emerge in single-return vs. chronically returned cats. 

While our total sample of cats was large (2642), the number of returns was low, 309 (11.70%). This is certainly a good thing for all those involved (including the shelter, cats, and owners), but it does make it harder to look at specific reasons why cats were returned, because once the sample is broken down, the number of cats in each category becomes quite small.

Lastly, while we did find significantly different risk ratios for reasons for return between long and short returns, some caution is warranted in the interpretation. Some of the confidence intervals of the risk ratios were very large because the number of cats was relatively small after being divided into each reason for return. Therefore, while there is certainly a significant difference in the likelihood of different types of returns between short and long returns, the magnitude of increased risk is not certain.

## 5. Conclusions

The return of shelter cats is a negative event for the owners, shelters, and, most of all, the cats. While tracing individual cats through the shelter system over multiple years is labor-intensive, this study design is also deeply informative. Because the return of cats to shelters is a multidimensional issue, there is a need for further exploration of intuitive and non-intuitive factors that impact the return of cats to shelters. By broadening our understanding of why cats are returned, and the timing of returns, it becomes easier to move towards more effective solutions.

## Figures and Tables

**Table 1:** Relative risk of different outcomes following a return in the short term (<30 days) and long term (>30 days). Bold indicates significant at the *p* < 0.05 level.

Outcome	Short Term (*n*)	Long Term (*n*)	Risk Ratio	CI	Z Score	*p*-Value
**Adoption**	**141**	**122**	**1.15**	**1.04, 1.27**	**2.86**	**0.004**
**Euthanasia**	**8**	**29**	**0.27**	**0.13, 0.58**	**3.38**	**0.008**
Transfer out	6	3	1.99	0.51–7.80	0.98	0.32

**Table 2:** Relative risk difference of common reasons for return in the short and long term. Bold indicates significant at the *p* < 0.05 level.

Reason for Return	Short Term (*n*)	Long Term (*n*)	Risk Ratio	95% CI	Z Score	*p*-Value
**Unwanted**	**11**	**1**	**10.93**	**1.43–83.63**	**2.30**	**0.02**
**Other pet**	**27**	**5**	**5.37**	**2.12–13.57**	**3.55**	**0.003**
Medical	18	9	1.99	0.92–4.29	1.75	0.08
**Behavior**	**50**	**29**	**1.71**	**1.15–2.55**	**2.64**	**0.008**
Other	4	4	0.99	0.25–3.90	0.009	0.99
Allergic	12	14	0.85	0.41–1.78	0.43	0.33
Housing	8	15	0.53	0.12–1.21	1.50	0.12
**Personal**	**14**	**28**	**0.50**	**0.27–0.91**	**2.28**	**0.02**
**Cost**	**7**	**18**	**0.39**	**0.16–0.90**	**2.21**	**0.03**
**Stray**	**3**	**17**	**0.18**	**0.05–0.59**	**2.83**	**0.005**
**Euthanasia**	**1**	**14**	**0.07**	**0.01–0.53**	**2.57**	**0.01**

## Data Availability

The data used in this study are available as a Supplemental File (S1).

## Supplementary Materials

The following are available online at https://www.mdpi.com/article/10.3390/ani13020243/s1, Data file S1: Supplemental data.
